# Fertilization altered co-occurrence patterns and microbial assembly process of ammonia-oxidizing microorganisms

**DOI:** 10.1038/s41598-022-26293-w

**Published:** 2023-05-22

**Authors:** Mingchao Ma, Yubin Zhao, Xin Jiang, Dawei Guan, Ming Yuan, Fengming Cao, Li Li, Jing Zhou, Jianli Ding, Jun Li

**Affiliations:** 1 Institute of Agricultural Resources and Regional Planning, Chinese Academy of Agricultural Sciences, Beijing, 100081 China; 2 Laboratory of Quality & Safety Risk Assessment for Microbial Products, Ministry of Agriculture, Beijing, 100081 China; 3 Qiqihar Sub-Academy of Heilongjiang Academy of Agricultural Sciences, Qiqihar, 161006 Heilongjiang China; 4 School of Life Sciences, Qufu Normal University, Jining, 273165 China; 5 Institute of Plant Nutrition and Resources, Beijing Academy of Agriculture and Forestry Sciences, Beijing, 100097 China

**Keywords:** Biophysical chemistry, Biodiversity, Community ecology, Microbial ecology

## Abstract

Ammonia-oxidizing archaea and bacteria (AOA and AOB, respectively) are important intermediate links in the nitrogen cycle. Apart from the AOA and AOB communities in soil, we further investigated co-occurrence patterns and microbial assembly processes subjected to inorganic and organic fertilizer treatments for over 35 years. The *amoA* copy numbers and AOA and AOB communities were found to be similar for the CK and organic fertilizer treatments. Inorganic fertilizers decreased the AOA gene copy numbers by 0.75–0.93-fold and increased the AOB gene copy numbers by 1.89–3.32-fold compared to those of the CK treatment. The inorganic fertilizer increased *Nitrososphaera* and *Nitrosospira.* The predominant bacteria in organic fertilizer was *Nitrosomonadales*. Furthermore, the inorganic fertilizer increased the complexity of the co-occurrence pattern of AOA and decreased the complexity pattern of AOB comparing with organic fertilizer. Different fertilizer had an insignificant effect on the microbial assembly process of AOA. However, great difference exists in the AOB community assembly process: deterministic process dominated in organic fertilizer treatment and stochastic processes dominated in inorganic fertilizer treatment, respectively. Redundancy analysis indicated that the soil pH, NO_3_^−^N, and available phosphorus contents were the main factors affecting the changes in the AOA and AOB communities. Overall, this findings expanded our knowledge concerning AOA and AOB, and ammonia‐oxidizing microorganisms were more disturbed by inorganic fertilizers than organic fertilizers.

## Introduction

Nitrogen is absorbed and utilized by microorganisms and crops that plays an essential role in regulating the global N cycle. Excessive application of nitrogen fertilizer has deeply changed soil N cycle and as one major factor resulted nitrogen loss. Ammonia oxidation, the key process of the soil N cycle, is chiefly carried out by ammonia-oxidizing bacteria (AOB), ammonia-oxidizing archaea (AOA) and complete ammonia oxidizing bacteria^1–3^. This further affected nitrogen leaching and retention with concomitant impacts on both crop productivity and the environment. Reducing inorganic fertilizer application and increasing organic fertilizer could alleviate the accumulation of soil nitrate nitrogen and improve the nitrogen utilization rate. Research on the microbiome of ammonia‐oxidizing microorganisms on different fertilizer application, as well as interaction mechanism, can help us comprehensively understand soil N cycling and dynamics, and formulate reasonable farmland fertilization strategies, which was of great importance in the reduce of chemical inputs and nitrogen loss.

Their abundance and distribution have been substantially researched in various habitats. However, little is known about the interaction mechanism of AOA and AOB corresponding to long-term fertilization. Studies have shown that excessive use of chemical fertilizers deleteriously changes the soil microbial community^4,5^. Intensive inorganic fertilizer inputs lead to a shift from fungal-dominated to bacterial soil food webs^6^, indicating that the capacity of material transformation was reduced^7,8^. The studies in our lab showed that soil bacterial diversity and abundance decreased due to the application of inorganic fertilization^9,10^. The AOA and AOB in black soil responded differently to environmental disturbances and resource utilization^11,12^. A 50-year field trial showed that the type and concentration of organic matter affected the structures of AOB and AOA communities in the clay loam soil^13^. Shi et al.^14^ demonstrated that organic fertilizers have significant effects on the diversity and community structure of AOB. All these findings indicate that inorganic and organic fertilizers have different properties, leading to questions about what aspects of AOA and AOB in the soil are influenced by the two fertilizer types and to what extent the AOA and AOB communities can be changed after the application of fertilizers.

In recent years, network analysis has become an accepted advanced research method to analyze the competition or mutually beneficial cooperation between many microbial communities, such as bacteria, fungi, actinomycetes and archaea^15,16^. The utility of network analysis accounts for potential biotic interactions between soil microorganisms that define the niche space of AOA and AOB^17^. The majority of nitrite-oxidizing bacteria and AOA formed two independent modules. Hence, changes in ammonia-oxidizing archaeal and bacterial co-occurrence patterns in soil undergoing long-term inorganic and organic fertilizer treatments needs to be investigated. The hub of AOA and AOB could provide further knowledge to understand the key connected microbe in the nitrification process and its mechanism in the northeast black soil.

The microbial assembly process, one of the advanced and prevalent approaches currently adopted in ecology for exploring microbial mechanisms, has obtained extensive research results in recent years^18–20^. The niche-based and neutral theories were adopted to perform quantitative analysis and examine the contributions of deterministic and stochastic processes in the microbial community assembly. While the niche-based theory is based on the differences in ecological niches of co-occurring species^21^, the neutral theory relies on dispersal and stochastic demographic processes^22^. Deterministic and stochastic processes simultaneously influence community assembly in various ecosystem types^23–25^. A recent study demonstrated that the assembly processes of AOA and AOB were different, and the balance between these two communities determined the species co-existence in the forest and meadow soils from temperate to tropical regions^26^. The AOA assembly processes were not altered by the conversion of grassland to cropland; however, the AOB community assembly process shifted from stochastic to deterministic processes in grassland and cropland, respectively^27^. This study has important implications for the potential diversification of soil functions under environmental changes. Therefore, assessing the long-term fertilizer application and how to differ the assembly process of ammonia-oxidizing microorganisms might provide a new strategy to control the nitrogen leaching and groundwater pollution in farmland.

The objective of this research was to comprehensively evaluate the interaction mechanism among ammonia-oxidizing microorganisms as a result of the introduction of inorganic and organic fertilizers in the black soil. We used quantitative polymerase chain reaction (qPCR) and high-throughput sequencing to detect the changes in AOA and AOB communities. Network analysis and microbial assembly process were used to assess the interactions among AOA and AOB affected by the inorganic and organic fertilizer. This study offers novel insights into the relationship between inorganic and organic fertilizers and ammonia-oxidizing microorganism.

## Results

### Soybean yield and soil properties

Fertilization improved soybean yield significantly, and the N1, N2 and M treatment increased the yield by 23.58%, 53.37%, and 39.55%, respectively, comparing with CK treatment (Fig. S1). Furthermore, there was no significant difference in yield among the organic (M) and the two inorganic treatments (N1 and N2), indicating that with an abundant nitrogen input, the organic fertilizer could support an ideal yield if used in place of the inorganic fertilizers.

The soil properties of the four treatments were different (Table 1). The pH of CK and M treatment was 6.48 and 6.59, and when the inorganic fertilizer was applied, the pH values of the N1 and N2 treated soils were reduced to 5.47 and 4.62, respectively. This finding indicated that the organic fertilizer could effectively maintain soil pH, whereas the inorganic fertilizer caused a pH reduction. There was more available nitrogen (NO_3_^−^-N and NH_4_^+^-N) in the fertilizer treatments than in the CK treatment, especially the NO_3_^−^-N content, which was higher by 115.90%, 138.16%, and 88.27% in the N1, N2, and M treatments, respectively, compared to that in the CK treatment. The N2 treatment had the highest AP content (64.85 mg kg^–1^), and the M treatment had the highest AK content (190.20 mg kg^–1^). The OM contents were higher in all fertilizer treatments than in the CK treatment.

**Table 1:** Soil physicochemical properties of the organic and inorganic fertilizer application.

Treatment	CK	N1	N2	M
pH	6.48 ± 0.06 a	5.47 ± 0.12 b	4.62 ± 0.28 b	6.59 ± 0.05 a
NN (mg kg^–1^)	2.36 ± 1.02 b	5.09 ± 0.45 a	5.62 ± 1.68 a	4.44a ± 0.62 a
AN (mg kg^–1^)	34.85 ± 0.57 a	48.44 ± 11.68 a	41.7 ± 9.32 a	37.47 ± 6.41 a
AP (mg kg^–1^)	2.89 ± 0.90 c	3.84 ± 0.67 c	64.85 ± 5.51 a	13.86 ± 0.98 b
AK (mg kg^–1^)	157.17 ± 29.27 b	185.1 ± 4.5 ab	174.33 ± 5.9 b	190.2 ± 1.48 a
TN (g kg^–1^)	1.18 ± 0.02 c	1.28 ± 0.08 b	1.39 ± 0.01 a	1.23 ± 0.06 b
OM (g kg^–1^)	24.46 ± 0.25 b	27.87 ± 0.75 a	27.79 ± 0.96 a	27.67 ± 0.12 a

Data are showed as mean (std. error).

### Copy numbers of *amoA* gene

Differences in the AOA and AOB *amoA* copy numbers among the treatments indicated that the sizes of the communities of ammonia-oxidizing microorganisms was influenced by long-term fertilization differently. In general, the AOA *amoA* copy numbers in CK and M treatment were much higher than that in N1 and N2; the AOB *amoA* copy numbers in CK and M treatment were much lower than that in N1 and N2 (Fig. S2). The copy numbers of AOA *amoA* gene were significantly lower by 75.15% in the N1 (7.99 × 10^6^ copies/g soil) and 93.33% in the N2 (2.15 × 10^4^ copies per ng DNA) treatments compared to those in the CK treatment. In contrast, the AOB *amoA* copy numbers in the N1 (1.86 × 10^4^ copies/g soil) and N2 (2.78 × 10^6^ copies/g soil) treatments were significantly (1.89 and 3.32-fold, respectively) higher than those in the CK treatment. The AOA and AOB *amoA* gene copy numbers in the M treatment (3.88 × 10^6^ and 5.37 × 10^5^ copies/g soil, respectively) were slightly lower, but not significantly different from the CK treatment gene copy numbers (AOA: 3.22 × 10^6^ and AOB: 6.44 × 10^5^ copies per ng DNA).

Correlation analysis among AOA and AOB sequence copy numbers and soil properties provided further insight into the factors driving the differences in AOA and AOB *amoA* copy numbers (Table 2). The AOA *amoA* copy numbers were negatively correlated with soil NO_3_^−^-N (r =  − 0.717, p < 0.01) and AP (r =  − 0.712, p < 0.01) levels and positively correlated with soil pH (r = 0.893, p < 0.01). However, the AOB *amoA* copy numbers were positively correlated with soil NO_3_^−^-N (r = 0.660, p < 0.05) and AP (r = 0.746, p < 0.01) concentrations and negatively correlated with soil pH (r =  − 0.868, p < 0.01). Therefore, soil pH, NO_3_^−^-N content, and AP content played vital roles in the changes in *amoA* copy numbers among all fertilizer treatments.

**Table 2:** Correlation analysis of α-diversity indices and *amoA* genes for ammonium-oxidizing microorganism and soil physicochemical properties.

		pH	NN	AN	AP	AK	TN	OM
AOA	*amoA* gene copy numbers	0.893**	− 0.717**	− 0.444	− 0.712**	0.383	0.073	− 0.366
	ACE	− 0.405	0.514	− 0.109	0.348	0.322	− 0.320	0.326
	Chao 1	− 0.319	0.553	− 0.125	0.449	0.216	− 0.361	0.176
	Shannon	− 0.339	0.467	0.353	− 0.233	0.459	− 0.766**	0.320
	Simpson	0.571	− 0.643*	− 0.438	0.018	− 0.316	0.666*	− 0.345
AOB	*amoA* gene copy numbers	− 0.868**	0.660*	0.492	0.746**	− 0.470	− 0.007	0.354
	ACE	0.128	0.363	0.053	0.258	− 0.047	− 0.336	0.146
	Chao 1	− 0.046	0.460	− 0.009	0.203	0.023	− 0.372	0.229
	Shannon	− 0.934**	0.559	0.469	0.584*	− 0.400	0.073	0.369
	Simpson	0.957**	− 0.544	− 0.496	− 0.501	0.399	− 0.066	− 0.316

* and **Correlation is significant at p < 0.05 and p < 0.01.

### Alpha diversity of soil AOA and AOB communities

The coverage, diversity and richness indices of the four fertilization treatments are presented in Table S1. The values of the community Chao1 and ACE indices were higher in the fertilizer treatments than those in the CK treatment for both AOA and AOB diversity. Moreover, the fertilizer treatments significantly affected the AOA diversity. The Chao1 index for M, N1 and N2 treatments increased by 15.16% to 19.99% for AOA diversity and by 2.42% to 9.51% for AOB diversity compared to that for the CK treatment. Furthermore, the AOA and AOB Shannon indices were higher for the fertilizer treatments than those for the CK treatment, except for the AOB Shannon index for the M treatment.

The relationships between the AOA and AOB alpha diversity and the soil properties are shown in Table 2. For the AOA, the Shannon indices were negatively correlated with soil TN content (r =  − 0.766, p < 0.01), and the Simpson indices were positively correlated with soil NO_3_^−^N (r =  − 0.643, p < 0.05) and TN (r = 0.666, p < 0.05) contents. In contrast, for the AOB, the Shannon indices were negatively correlated with soil pH (r =  − 0.934, p < 0.01) and positively correlated with soil AP levels (r = 0.584, p < 0.05), and the Simpson indices were positively correlated with soil pH (r = 0.957, p < 0.01). These results indicated that alpha diversity of AOA was more sensitive to soil NO_3_^−^-N content, whereas alpha diversity of AOB was more sensitive to soil pH.

### Beta diversity of the AOA and AOB communities

**Figure 1:**
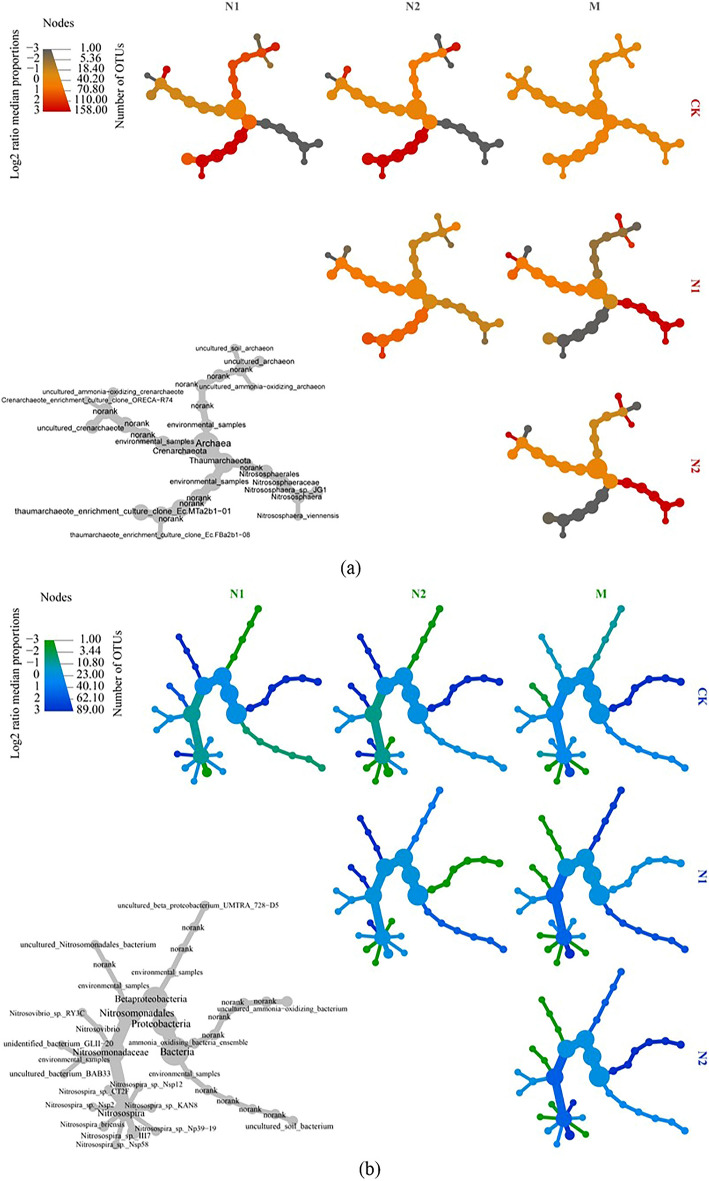
Heat tree of ammonia-oxidizing archaea (AOA) and ammonia-oxidizing bacteria (AOB) followed by inorganic fertilizer and organic fertilizer application in black soil.

Analysis revealed that the types of microbes observed were similar across the treatments, but the microbial abundances differed (Fig. S3). The predominant phyla in the AOA communities in the four treatments included Thaumarchaeota, Archaea_unclassified, Crenarchaeota, and environmental_samples. Environmental_samples_norank, Archaea_unclassified*,* and Nitrososphaera were the dominant genera in the four fertilization treatments (Fig. S3a,b). The composition of the AOA communities in the soils of the M and CK treatments was largely similar, and the predominant archaeal genera were *Environmental_samples_norank* (64.51% and 68.43%, respectively) and *Archaea_unclassified* (35.44% and 31.53%, respectively). The predominant archaea in the N1 and N2 treatment soils differed from those in the CK treatment soil. The abundance of *Environmental_samples_norank* in the N1 and N2 treatment soils was 77.11% and 84.29% lower, respectively, than that in the CK treatment, and the abundant archaeal genus *Nitrososphaera* was significant enrichment in N1 (23.39%) and N2 (46.60%) (Fig. 1a).

The predominant phyla in the AOB communities in the four treatments included the Proteobacteria, Bacteria_unclassified, and environmental_samples (Fig. S3c,d). The composition of the AOB communities in the M and CK treatment soils was largely similar, and the enrichment bacteria were *Nitrosomonadales_unclassified* (54.58% and 62.24%, respectively), and *Bacteria_unclassified* (7.25% and 7.20%, respectively). The predominant bacteria in the N1 and N2 treatment soils were significantly different from those in the CK treatment soils. The most abundant bacterial genus was *Nitrosospira* (N1: 77.57% and N2: 82.63%), followed by *Bacteria_unclassified* (N1: 15.90% and N2: 7.68%) (Fig. 1b). In the N1 and N2 treatment soils, the abundance of *Nitrosospira* was 1.20-fold higher, *Bacteria_unclassified* was 1.19-fold and 5.93% higher, respectively, compared to that in the CK treatment soils.

### Correlations between soil properties and AOA and AOB community

**Figure 2:**
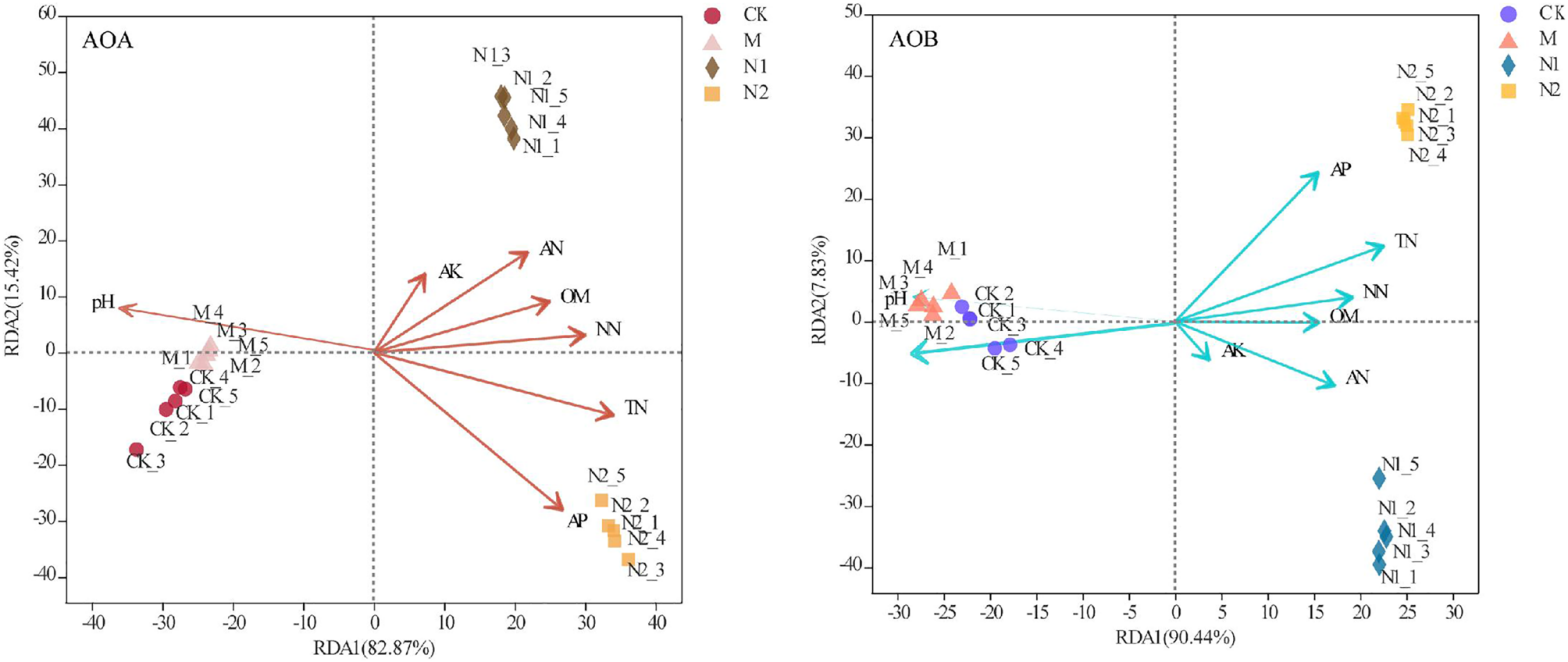
Redundancy analysis (RDA) of the AOA and AOB community structure and relationships with soil factors.

Redundancy analysis (RDA) of the unweighted UniFrac distance between samples revealed a strong degree of clustering of established AOA communities in relation to the four treatments (Fig. 2). All samples were well separated along the PC1 axis for both AOA and AOB, which explained 82.87 and 90.44% of the total variation. Organic and inorganic fertilizer treatment were distinguished in two clusters: one cluster included the CK and M treatments, whereas the other cluster comprised N1 and N2 samples. Moreover, the N1 and N2 clusters resolved along the PC2 axis and explained 15.42% and 7.83% of the total variation for AOA and AOB.

**Figure 3:**
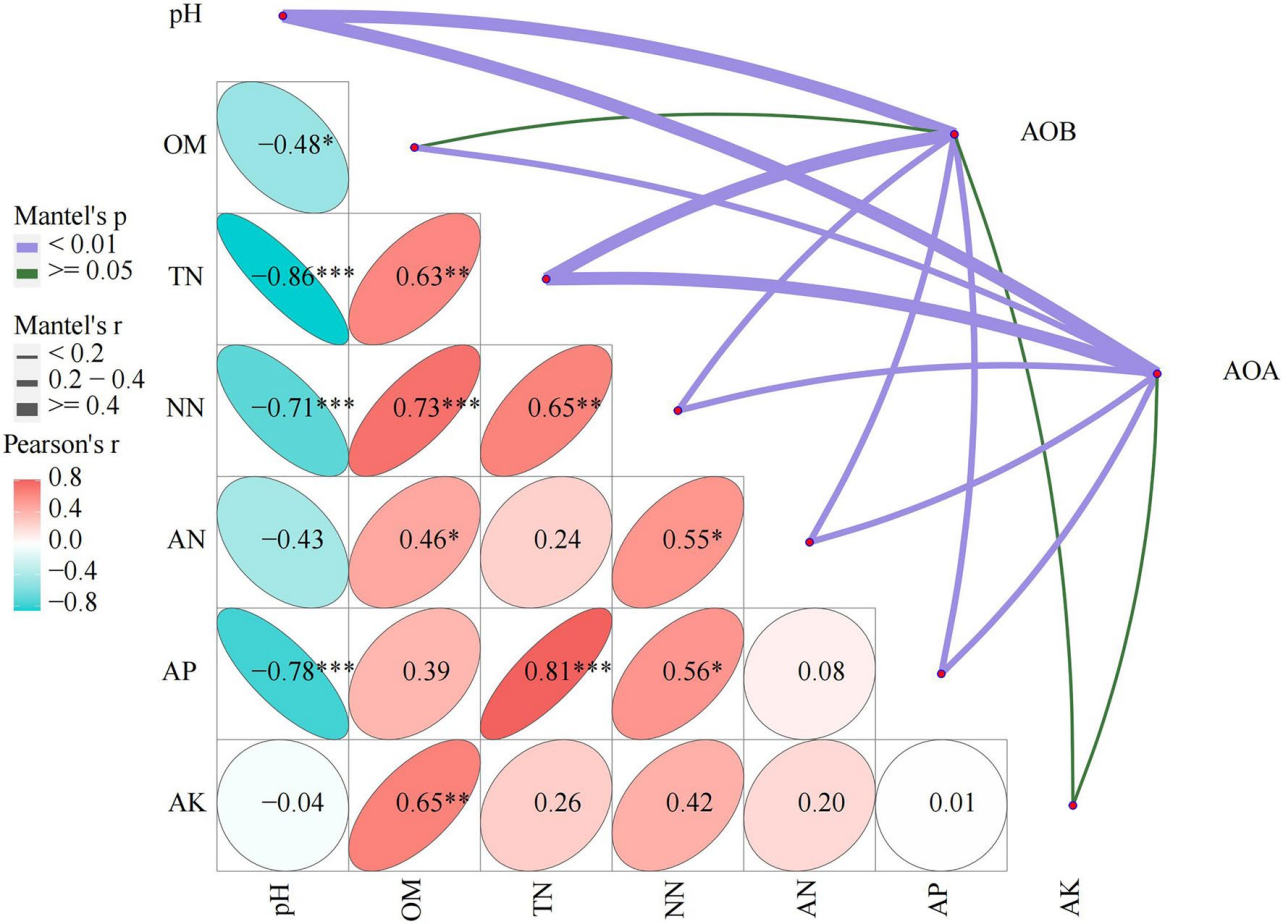
Pairwise comparisons of environmental factors are shown, with a color gradient denoting Spearman’s correlation coefficients. AOA and AOB community composition was related to each environmental factor by partial (geographic distance-corrected) Mantel tests. Edge width corresponds to the Mantel’s r statistic for the corresponding distance correlations, and edge color denotes the statistical significance.

RDA also showed that the variation in the AOA and AOB community structure could be explained by all seven of the soil properties. The AOA and AOB community of organic fertilizer were changed mainly by the soil pH. The inorganic fertilizer treatments changed by the different soil properties. The N2 treatment showed a positive correlation with the AP and TN content, and the N1 treatment showed a positive correlation with AN and AK. The mantel test showed (Fig. 3, Table S2) that the main contributor to the differences in AOA community structures was soil pH (r = 0.89, p < 0.05),, and the secondary contributors to the AOA community structure differences were the TN (r = 0.61, p < 0.05), NN (r = 0.40, p < 0.05) and AP (r = 0.40, p < 0.05) contents. The soil properties affected the AOB community structure according to the order (P < 0.05): pH (r = 0.87) > TN (r = 0.56) > AP (r = 0.33).

### Co-occurrence pattern of soil AOA and AOB

**Figure 4:**
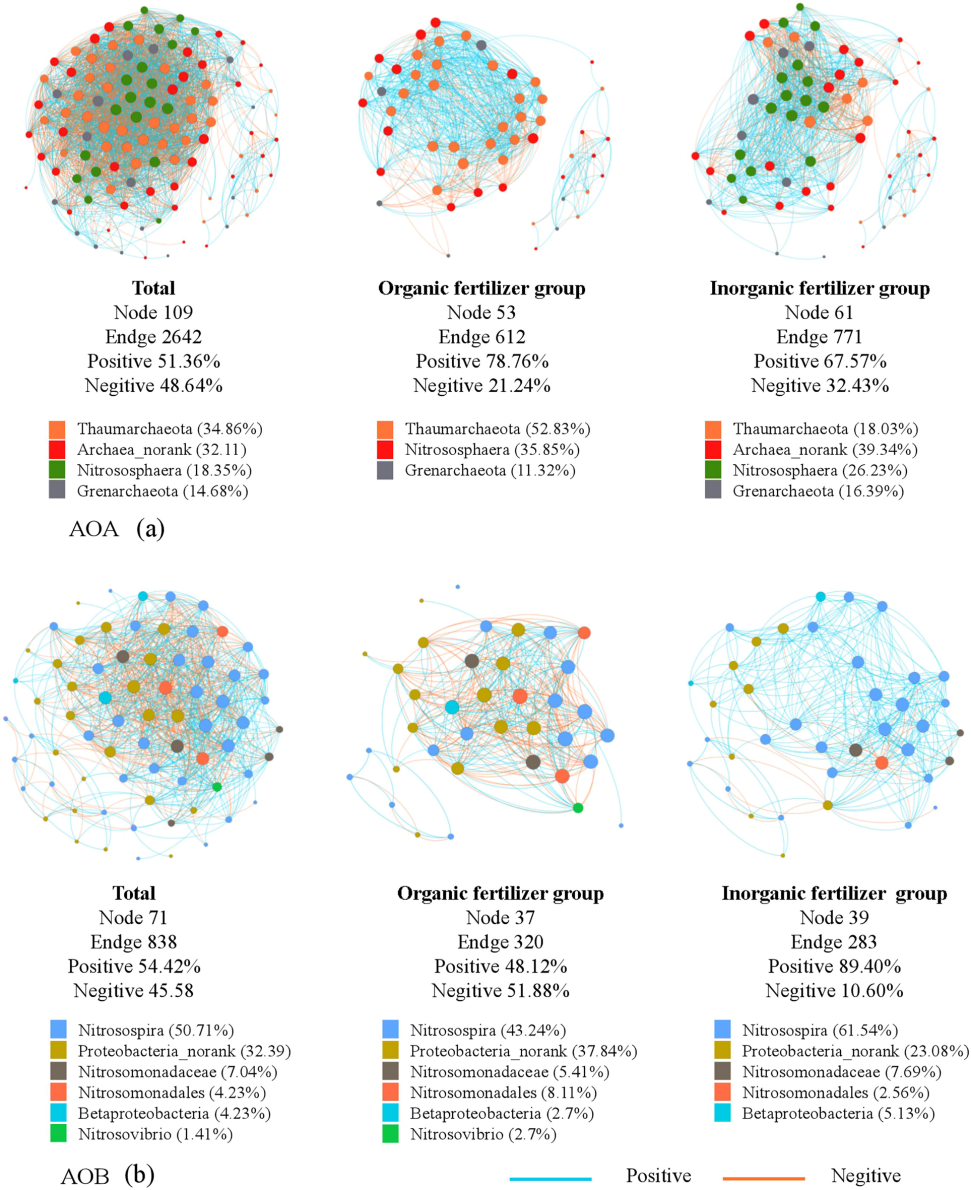
The networks visualized inorganic fertilizer and organic fertilizer treatment effects on the co-occurrence pattern between AOA (**a**) and AOB (**b**) taxa at operational taxonomic unit (OTU) level.

To evaluate the impact of the different fertilizer treatments on microbial associations, six networks at the OTU level were constructed for the AOA and AOB, respectively (Fig. 4). Overall, the co-occurrence of soil AOA communities was more complex than that of the AOB communities, and fertilizer application caused differences in the co-occurrence patterns of the AOA and AOB. The number of nodes for AOA in networks of the inorganic fertilizer group was higher by 15.09%, and the number of edges was higher by 25.98%, respectively, compared to those of the organic fertilizer group. The number of nodes for AOB in networks of the inorganic fertilizer group was higher by 5.41%, and the number of edges was lower by 11.56%. compared to those of the organic fertilizer group. The positive correlation coefficients also differed, and the inorganic fertilizer group had lower values than the organic fertilizer group for AOA but higher values than the organic fertilizer group for AOB. These results suggest that the amount of mutually beneficial cooperation in AOB communities increased under organic fertilizer group; however, there was an increased level of competition in AOA communities in organic fertilizer group.

Hub analysis further suggested that the AOA and AOB taxa differed greatly among the organic and inorganic fertilizer groups. We selected the top 10 microbial taxa for this study (Tables S3,S4). The diversity of both the hubs AOA and AOB increased in the inorganic fertilizer group than that in the organic fertilizer group. Most of the connected hub AOA and AOB in the organic fertilizer group were Thaumarchaeota and *Nitrosospira*. The connected hubs AOA and AOB in the inorganic fertilizer group included Thaumarchaeota, *Nitrosospira*, *Nitrososphaera,* and unranked Proteobacteria. The different key hub operational taxonomic units (OTUs) in both AOA and AOB taxa indicated that the inorganic and organic fertilizers changed the dependence of connected microbial species. The data on the network module class also supported these results, and the hub OTUs in the inorganic and organic fertilizer groups belonged to different module classes.

### Microbial assembly process of AOA and AOB in soil

**Figure 5:**
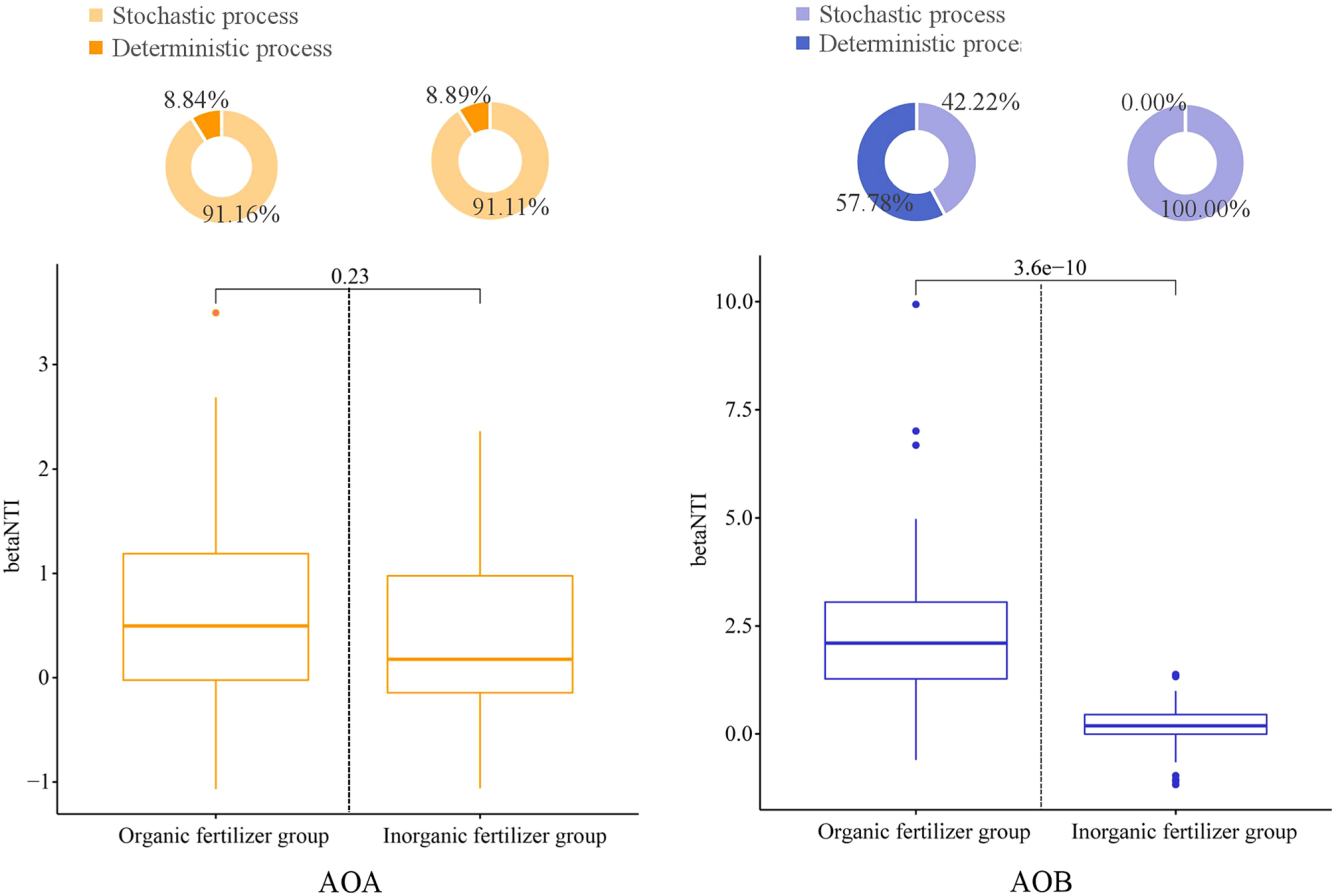
The community assembly processes by fitting niche-based theories. Pie plots show the relative contribution of stochastic and deterministic process in AOA and AOB community assembly following with the long-term inorganic fertilizer and organic fertilizer application.

**Figure 6:**
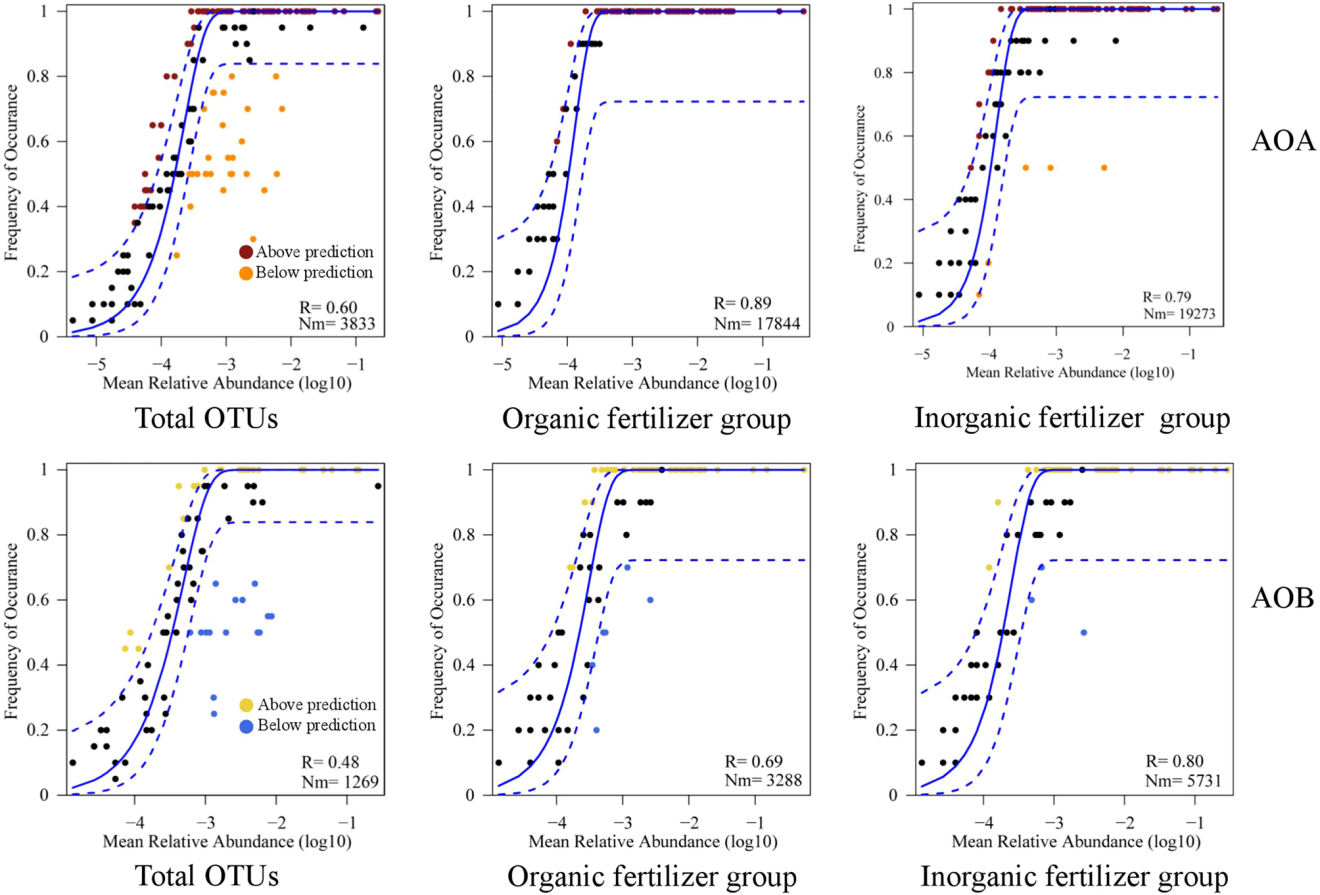
Neutral community model (NCM) of AOA and AOB community assembly. The predicted occurrence frequencies for inorganic fertilizer and organic fertilizer, and all representing soil AOA and AOB communities. The solid blue lines indicate the best fit to the NCM, and the dashed blue lines represent 95% confidence intervals around the model prediction. OTUs that occur more or less frequently than predicted by the NCM are shown in different colors. Nm indicates the metacommunity size times immigration, and R indicates the fit to the model.

The obtained betaNTI value indicated that stochastic processes were crucial in shaping the AOA community assembly, and the relationships between the occurrence frequency of OTUs and their relative abundance variations were 69% and 80%. We acknowledge that the organic fertilizer negligibly increased the stochastic processes for the AOA community assembly and significantly increased the deterministic processes for the AOB community assembly compared to the inorganic fertilizers (Fig. 5). The NCM successfully estimated a large fraction of the relationship between the occurrence frequency of OTUs and their relative abundance variations (Fig. 6), with 89% and 79% of explained AOA community variance for the organic fertilizer group and inorganic fertilizer group, respectively. The Nm-value was higher for both AOA (Nm = 19,273) and AOB (Nm = 5731) with the inorganic fertilizer group than that of the organic fertilizer group (Nm = 17,844; Nm = 3288). These results indicate that inorganic fertilizer increased the species diffusion of AOA and AOB.

## Discussion

### Changes in the abundance of the AOA and AOB

AOA are known to be more suitable than AOB in acidic soils, while AOB are more active in alkaline and neutral environments^28^. However, some studies have identified AOB as the dominant ammonia oxidizers in several acidic soils^29^, and play a major role in acidic soil nitrification^30–32^. Moreover, AOB can exhibit high nitrification rates under a soil pH of 3^33^. From the correlation analysis, we established that soil pH was the main factor affecting the AOA and AOB *amoA* copy numbers, similar to the results of Li et al.^34^ and Singh et al.^35^. We speculate that the differences in the results are due to a variation in the relative abundances of AOA and AOB from fractional to several orders of magnitude depending on factors in addition to soil pH, including organic matter, nitrogen content and other environmental factor^36^. In our study, long-term fertilization with inorganic fertilizers reduced the growth of AOA but increased the growth of AOB in black soil compared to that with organic fertilizers. This results was similar to the obvious research, which indicated that showed that chemical fertilizers can decrease the diversity of the AOA community^2,37^. Organic fertilizer provides a variety of inorganic and organic nutrients, such as organic acids, for crops while fertilizing the soil, maintaining soil pH and improving the soil structure and ecosystem^38,39^. The VPA in our study showed that soil pH explained 78.09% and 60.58% and TN explained 60.58% and 53.09% of the observed changes in the AOA and AOB communities, respectively (Table 3). This suggests that the high soil N content and the relatively low pH of soils were the two main reasons leading to the differences in the AOA and AOB communities after inorganic fertilizer application in black soil. Increasing the soil pH and providing more complex nutrients (e.g., amino acid content) may be the reason for maintaining the soil AOA and AOB community after organic fertilizers application^40^.

**Table 3:** Variation partitioning analysis (VPA) of AOA and AOB β-diversity variance explained by soil physicochemical properties.

	R^2^	
	AOA (%)	AOB (%)
pH	78.09	85.55
TN	60.58	53.09
NN	44.70	35.71
AP	42.92	27.00
OM	29.76	21.21
AN	24.13	28.51
AK	− 0.82	− 3.73
Residuals	2.06	2.26

A pot experiment showed that *Nitrosospira* (AOB) and *Nitrososphaera* (AOA) dominated absolutely in organic fertilizer treatments in which the soil pH was 7.8–8.4^13^. In our study, *Nitrosospira* and *Nitrososphaera* were the dominant microorganisms in the soils of the inorganic fertilizer group, which had relatively low pH values (5.47–5.62), but not in the soils of the organic fertilizer group, which had higher pH values (6.48–6.59). This phenomenon is not only related to the physical and chemical properties of the soil but also the acid sensitivity, substrate utilization, and other characteristics of microbes. Previous research showed that *Nitrosospira* of AOB play a vital role in the nitrification process in acidic soil following both long-term inorganic and organic fertilization^41^. After isolation, some *Nitrosospira* spp. were possibly involved in the ammonium oxidation at low soil pH of 4^42^. We speculate this as a reason for the abundance of *Nitrosospira* in the inorganic fertilizer treatment in our study. Advanced technology is required to isolate additional strains of AOB that cannot be cultivated in the experiment and confirm the above. In addition, Zhalnina et al.^43^ showed that the abundance of *Nitrososphaera* negatively correlates with the abundance of bradyrhizobia. The *Nitrosomonadales*, which are beneficial for plant growth and inhibition of pathogens causing root rot^44,45^, were abundant in the organic fertilizer in our study. Therefore, for the development of sustainable agriculture, we must thoroughly consider microbial changes and adjust the fertilization strategy, e.g., usage of organic fertilizers instead of chemical fertilizers, to avoid microbial disorders.

### Changes in the co-occurrence pattern of the AOA and AOB

Co-occurrence patterns are helpful for evaluating changes in microbial community structure and provide insights into potential microbial interactions^46^. Banerjee et al.^47^ indicated that intensive agricultural production could decrease the network complexity of bacteria. The corresponding positive and negative correlation coefficients further showed that intraspecific competition for AOA and mutual benefit symbiosis for AOB were increased in the inorganic fertilizer treatments. Network analysis could define the niche space of microorganism communities for soil management^48^. Inorganic fertilizer increased the network complexity of AOA but decreased the network complexity of AOB that can be attributed to the high eco-physiological diversity and survival in a wider habitat of AOB^13^. Fortunately, we also found that chemical fertilizers can indeed significantly increase the niche width of AOB and negligibly increase the niche width of AOA compared with that by organic fertilizers (Fig. S4). Therefore, after inorganic fertilizer application, the AOB community occupied more ecological niches and consumed more resources, resulting in a decreasing connection. To increase the ability of competitiveness with AOB for more resources, the AOA community need more cooperation, resulting in the increasing connection after inorganic fertilizer. Moreover, the hub *Nitrososphaera* increased significantly in the network, and some important Proteobacteria were missing after inorganic fertilizer application. This shift might result the functional changes in the nitrogen cycle and microbial assembly process of the AOA and AOB.

### Changes in microbial assembly process of the AOA and AOB

Compared to organic fertilizer, inorganic fertilizer affects the microbial assembly process of AOB more than AOA. Research has shown that when the pH decreases, ammonia (NH_3_) is converted into ammonium (NH_4_^+^), affecting the acquisition of AOB, thereby increasing the number and activity of AOB^49^. Rütting et al.^50^ indicated that the contribution to ammonia oxidation of AOB outcompeted AOA under higher ammonium supply. The results are in accordance with environmental data suggesting that AOA are mainly responsible for ammonia oxidation under more oligotrophic conditions, whereas AOB dominate under eutrophic conditions^51^. When the AOB community has sufficient resource to survive, the structure of the AOB community is not easily affected by soil physicochemical properties (e. g. NH_4_^+^); however, it is affected by the birth and death of microorganisms. Thus, the microbial assembly process of AOB changed from a deterministic process in organic fertilizer to a stochastic process in inorganic fertilizer. However, AOA possess much higher substrate affinities than the comammox or AOB counterparts^52^, resulting in the microbial assembly process of AOA being less susceptible to substrate changes from inorganic fertilizer application.

Assembly mechanism could be one of the key processes in shifting the microbial functions. When deterministic assembly dominates the assembly process, a higher diversity of the community would generally show better reactor performance, and when the stochasticity dominates the assembly process, the functional performance declines^1^. In our study, we found that inorganic fertilizer could highly increase aerobic ammonia oxidation, nitrification, and ureolysis process compared to that with organic fertilizer (Fig. S5). This might be the reason for organic fertilizer having more *amoA* genes of AOA but lower functional reads than inorganic fertilizer when they are under the stochastic assembly process.

Therefore, inorganic fertilizer changes the abundance of AOA and AOB and also affect the interaction and strengthens the nitrosation process of soil ammonia-oxidizing microorganism. Surely, additional nitrosation processes may lead to loss of nitrogen and contamination of groundwater caused by excessive application of inorganic fertilizers. There is still much work to be explored to develop sustainable agriculture by maintaining a dynamic balance between fertilizer application, crop yield, and ecological stability.

In conclusion, organic and inorganic fertilizer distinctly alter the abundance, co-occurrence pattern, and microbial assembly process of soil AOA and AOB differently. Inorganic fertilizer decreased the *amoA* copy numbers of AOA and increased the *amoA* copy numbers of AOB. At the same time, compared with organic fertilizer, inorganic fertilizer could imply an ecological imbalance among AOA and AOB communities in enriching *Nitrososphaera* and *Nitrosospira* and decreasing *Nitrosomonadales*. Changes in soil AOA and AOB community structure and *amoA* copy numbers were primarily due to shifts in the low pH and high NN and AP content by application of inorganic fertilizer. Moreover, inorganic fertilizer increased the complexity of the co-occurrence pattern of AOA but decreased the complexity pattern of AOB. Our results also suggest that the inorganic fertilizer has little effect on the microbial assembly process of AOA, whereas the application of inorganic fertilizer significantly increases the stochastic process of AOB. Overall, organic fertilizer has less disturbance for AOA and AOB communities by maintaining neutral soil pH and alleviate the accumulation of NO_3_^−^N content. This study provides critical data for understanding the influence of inorganic and organic fertilizers on the soil ammonia-oxidizing archaeal and bacterial ecosystem, which will provide robust evidence to facilitate reasonable farmland fertilization strategies.

## Materials and methods

### Experimental design

The trial site located in Harbin, Heilongjiang Province, China (N 45° 40′, E 126° 35′). The four fertilization treatments assessed were CK (no fertilizer); N1 (low inorganic fertilizer), N2 (high inorganic fertilizer), and M (organic fertilizer, horse manure). The inorganic fertilizers used were urea, calcium super phosphate, and ammonium hydrogen phosphate. The dose of horse manure as an organic fertilizer was approximately 18,600 kg manure/ha. Fertilizer application details are provided in Table S5. The dose of fertilizer in this study referred to local fertilization level in northeast china.

### Black soil samples

We collected black soil samples from a 35-year positioning trial filed located in Harbin, Heilongjiang Province (N 45° 40′, E 126° 35′), which is one of the experimental stations belonging to China’s agricultural research system. During soybean harvest, we collected rhizosphere soil samples at 10 points by randomly extracting 20–25 bean individuals in each plot. Soil which loosely adhered to the roots were gently shaken off and the soil tightly adhered to roots were brushed with a brush, and mixing to obtain one composite soil sample. Five samples of each treatment and a total of 20 samples were collected, and all the soil samples were transported to the lab on ice. Some soil samples in each treatment were selected randomly and air-dried in the shade for analysis of physical and chemical properties. Some soil samples were randomly selected and stored at − 80 °C for analysis of microorganism.

### Soil properties

The soil pH was measured using a pH meter, which the ratio of soil and water was 1:1^53^. Kjeldahl method was used to detect the soil TN, and NO_3_^−^-N and NH_4_^+^-N content were measured by flow injection analysis^54^. Mo‐Sb colorimetric method was used to determine the soil AP level^55^. The soil AK content was evaluated by Flame photometry. Soil OM content was measured by the K_2_Cr_2_O_7_ capacitance method^56^.

### Soybean yield

Dry weight of soybeans harvested from a 10 m^2^ area in the central part of each plot was determined to calculate the soybean yield^57^.

### DNA extraction and qPCR

Total DNA of soil microorganism was extracted with a Power Soil DNA Isolation Kit (MOBIO Laboratories Inc., Carlsbad, CA, USA) and was stored at − 80 °C until further analysis. The AOB primers used were *amoA*-1F and *amoA*-2R^58^. The AOA primers used were *Arch-amoA-*F and *Arch-amoA-*R^59^. The ABI 7500 Real‐Time PCR Detection System was used to survey the number of *amoA* genes (Applied Biosystems, Waltham, MA, USA). The 20 μL PCR reaction system contained 10 μL UltraSYBR Mixture (CWBIO, China); 2 μL cDNA template of AOA or AOB; 0.2 μL forward and reverse primers (10 μM); and 7.6 μL ddH_2_O. The qPCR amplification cycle for AOA comprised the following: 94 °C 5 min, followed by 40 cycles of denaturation at 94 °C for 30 s, annealing at 63 °C for 40 s, and extension at 72 °C for 40 s. The qPCR amplification cycle for AOB comprised the following: 94 °C 5 min, followed by 40 cycles of denaturation at 95 °C for 60 s, annealing at 53℃ for 60 s, and extension at 72 °C for 90 s.

### Sequencing data processing

The primers used were the same as those used for qPCR. The total 25 μL PCR reaction system contained 40 ng of DNA template; 1 μL forward and reverse primers (10 μM); 0.25 μL Q5 high-fidelity DNA polymerase; 5.0 μL 5× High GC Buffer; 5.0 μL 5× Reaction Buffer; 0.5 μL dNTP (10 mM). The PCR amplification cycle comprised the following: 30 s at 98 °C, followed by 27 cycles of denaturation at 98 °C for 15 s, annealing at 58 °C/55 °C (AOA/AOB) for 30 s, and extension at 72 °C for 30 s/45 s (AOA/AOB), with a final elongation step for 7 min at 72 °C. The PCR products were used to purify and generate the amplicon libraries, following which the data were analyzed using QIIME pipeline version 1.8.0. A total of 255,338 and 322,885 sequences (more than 10,000 sequences per individual soil sample) for AOA and AOB, respectively, were obtained after quality trimming (Tables S3,S4). Operational taxonomic units (OTUs) were defined by clustering at 97% similarity. The α‐diversity of AOA and AOB was calculated using mothur^60^ and with the following four parameters as richness indices: Shannon and Simpson diversity, Chao 1 and the abundance‐based coverage estimator (ACE). The pyrosequencing data could be detected in the NCBI by SRA accession (PRJNA51207; SRX1034826).

### Statistical analysis

Interactions between the AOA and AOB communities and environmental factors were analyzed using CANOCO 5.0 for redundancy analysis (RDA). The network analysis was conducted at the OTU level of AOA and AOB for all the total 20 sample, and visualized with Gephi (version 0.9.2). Totally, 158 archaeal and 89 bacterial OTUs were analyzed. We calculated all pair-wise spearman correlations between OTUs by SPSS 20. The Spearman’s correlations |*r*|> 0.8 and P value of less than 0.01 were retained^61^. Moreover, among all the samples, according the RDA analysis, the samples were divided into two group: CK and M; N1 and N2. Thus, the two group were selected for the sub-network analysis. Sub-networks were produced from the total network by preserving the presented nodes and edges^62^. The topological parameters of the network were also computed using Gephi (version 0.9.2). We adopted a neutral community model (NCM) to predict the relationship between OTU detection frequencies and their relative abundance across the wider metacommunity, which were performed using R (version 3.6.3) and the program presented by Chen et al.^20^. Moreover, the assembly processes of AOA and AOB communities were evaluated by calculating the nearest taxon index and beta nearest taxon index (betaNTI) using the “ses.mntd” function in a picante package^63,64^. The contribution was considered a stochastic process when |betaNTI|< 2, and the shifts in community composition were deterministic processes when |betaNTI|> 2. The retained resulting correlations were imported into the Gephi platform to obtain the topology property parameters for the network^65^. Functional prediction was analyzed through FAPROTAX (http://www.cloud.biomicroclass.com). Soil physical and chemical property data were analyzed using Microsoft Excel 2010 pro and SPSS version 20.

### Ethical approval

This article does not contain any studies with human participants or animals performed by any of the authors. This study complies with relevant institutional, national, and international guidelines and legislation. We declare that we have the appropriate permissions for collection of plant or seed specimens.

## Supplementary Information

Supplementary Information.

## Data Availability

The datasets used and analyzed during the current study available from the corresponding author on reasonable request.
