# Supplementary material for: Fertilization altered co-occurrence patterns and microbial assembly process of ammonia-oxidizing microorganisms
Source: Sci Rep. 2023 May 22;13:8234. doi: 10.1038/s41598-022-26293-w (PMC10202944; doi:10.1038/s41598-022-26293-w)
Supplement: Supplementary file 1 — Supplementary Information. [file 41598_2022_26293_MOESM1_ESM.docx]

**Fertilization altered co-occurrence patterns and microbial assembly process of ammonia-oxidizing microorganisms**

Mingchao Ma^1,2†^, Yubin Zhao^1,2†^, Xin Jiang^1,2*^, Dawei Guan^1,2^, Ming Yuan^3^, Fengming Cao^1,2^, Li Li^1,2^, Jing Zhou^4^, Jianli Ding^5^, Jun Li^1,2*^

^1^ Institute of Agricultural Resources and Regional Planning, Chinese Academy of Agricultural Sciences, Beijing, 100081, China

^2^ Laboratory of Quality & Safety Risk Assessment for Microbial Products, Ministry of Agriculture, Beijing, 100081, China

^3^ Qiqihar Sub-academy of Heilongjiang Academy of Agricultural Sciences, Qiqihar, Heilongjiang, 161006, China

^4^ School of Life Sciences, Qufu Normal University, Jining, 273165, China

^5^ Institute of Plant Nutrition and Resources, Beijing Academy of Agriculture and Forestry Sciences, Beijing, 100097, China

†Mingchao Ma and Yubin Zhao contributed equally to this work.

1

*Corresponding Author: [lijun01@caas.cn](mailto:lijun01@caas.cn), [jiangxin@caas.cn,](mailto:jiangxin@caas.cn,) Institute of Agricultural Resources and Regional Planning, Chinese Academy of Agricultural Sciences, Beijing 100081, PR China. Tel: +8610 82106208. FAX: +86 1082108702

*Corresponding Author: [lijun01@caas.cn](mailto:lijun01@caas.cn), jiangxin@caas.cn

Institute of Agricultural Resources and Regional Planning, Chinese Academy of Agricultural Sciences, Beijing 100081, PR China.

Tel: +8610 82106208.

FAX: +86 1082108702

Data availability: The datasets used and/or analysed during the current study available from the corresponding author on reasonable request.

**Fig. S1.** Soybean yield for the four fertilization treatments.


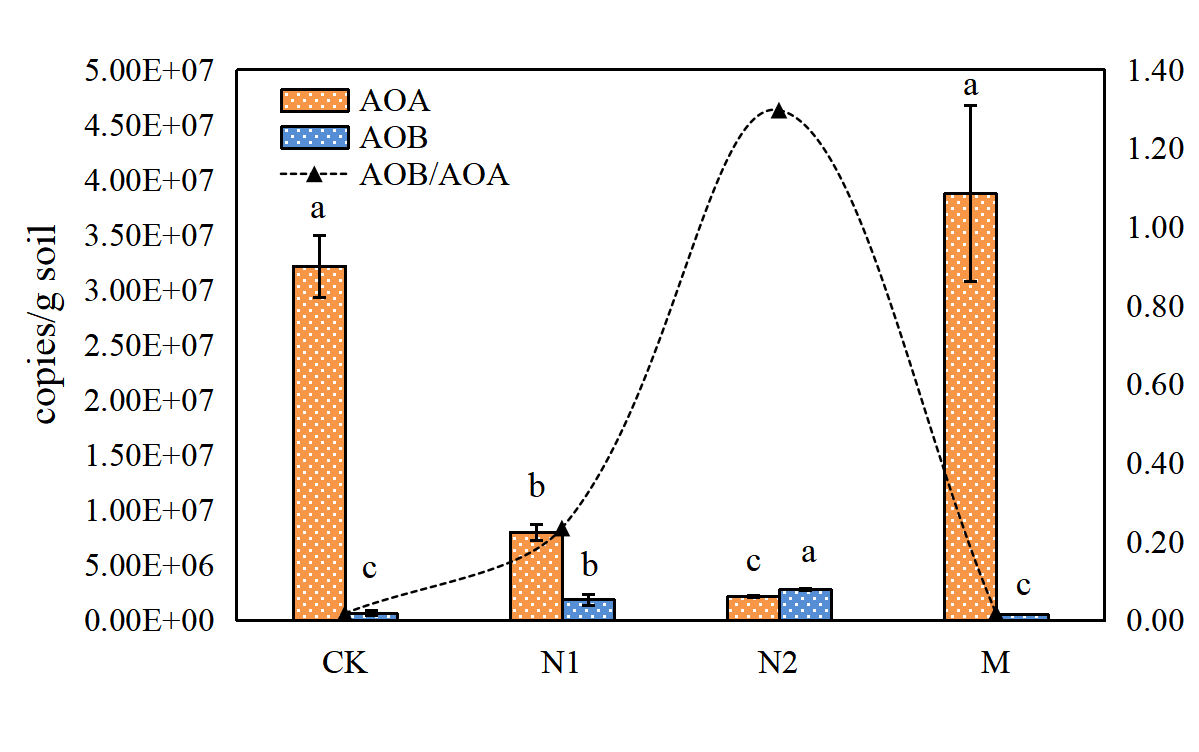


**Fig. S2.** Ammonium-oxidizing archaea (AOA) and ammonium-oxidizing bacteria (AOB) *amoA* copy numbers in soil samples from the four fertilization treatments.

**Fig. S3.** Community composition of soil microcosms established in archaeal (a, b) and bacterial (c, d) communities at the phylum and genus levels in soils of the four fertilization treatments.

**Fig. S4** Niche of width for AOA and AOB followed by inorganic fertilizer and organic fertilizer in black soil.

**Fig S5** Abundance of AOA and AOB of the functional prediction by FAPROTAX.

**Table S1** Diversity indices and coverage of AOA and AOB in black soils treated with organic and inorganic fertilizers for 35 years.

|  | Treatment | Ace | Chao 1 | Shannon | Simpson | Coverage |
| --- | --- | --- | --- | --- | --- | --- |
| AOA | CK | 99.67±4.74 b | 98.69±5.72 b | 2.33±0.10 c | 0.24±0.03 a | 0.999 |
|  | N1 | 112.94±6.71 a | 114.69±10.88 a | 2.61±0.02 a | 0.15±0.01 c | 0.999 |
|  | N2 | 116.78±7.56 a | 118.42±7.05 a | 2.41±0.08 c | 0.20±0.02 b | 0.999 |
|  | M | 111.64±5.87 a | 113.65±8.75 a | 2.50±0.01 b | 0.20±0.00 b | 0.999 |
| AOB | CK | 57.93±4.52 b | 57.39±6.14 a | 1.87±0.05 b | 0.32±0.02 b | 0.999 |
|  | N1 | 57.28±2.78 ab | 58.78±4.07 a | 2.18±0.05 a | 0.18±0.01 c | 0.999 |
|  | N2 | 61.56±2.80 ab | 62.85±5.09 a | 2.24±0.04 a | 0.17±0.00 c | 0.999 |
|  | M | 62.26±2.94 a | 62.83±1.64 a | 1.74±0.07 c | 0.37±0.02 a | 0.999 |

**Table S2.** Mantel test examine the relationships between soil physicochemical properties and soil AOA and AOB community in black soil.

| Soil Properties | AOA | | AOB | |
| --- | --- | --- | --- | --- |
|  | Mantel *r* | *p* | Mantel *r* | *p* |
| pH | 0.89 | 0.00 | 0.87 | 0.00 |
| OM (mg/kg) | 0.27 | 0.01 | 0.09 | 0.09 |
| TN (%) | 0.61 | 0.00 | 0.56 | 0.00 |
| NN (mg/kg) | 0.40 | 0.00 | 0.23 | 0.00 |
| AN (mg/kg) | 0.27 | 0.00 | 0.26 | 0.01 |
| AP (mg/kg) | 0.40 | 0.00 | 0.33 | 0.00 |
| AK (mg/kg) | 0.03 | 0.27 | -0.02 | 0.69 |

|  | OTU | Phylum | Class | Order | Family | Genus | Degree | Modularity class |
| --- | --- | --- | --- | --- | --- | --- | --- | --- |
| Organic fertilizer group | OTU9 | Thaumarchaeota | Thaumarchaeota | Thaumarchaeota | Thaumarchaeota | Thaumarchaeota | 37 | 0 |
|  | OTU86 | Thaumarchaeota | Thaumarchaeota | Thaumarchaeota | Thaumarchaeota | Thaumarchaeota | 37 | 0 |
|  | OTU108 | Thaumarchaeota | Thaumarchaeota | Thaumarchaeota | Thaumarchaeota | Thaumarchaeota | 36 | 0 |
|  | OTU75 | Thaumarchaeota | Thaumarchaeota | Thaumarchaeota | Thaumarchaeota | Thaumarchaeota | 35 | 1 |
|  | OTU98 | Thaumarchaeota | Thaumarchaeota | Thaumarchaeota | Thaumarchaeota | Thaumarchaeota | 35 | 1 |
|  | OTU18 | Thaumarchaeota | Thaumarchaeota | Thaumarchaeota | Thaumarchaeota | Thaumarchaeota | 33 | 1 |
|  | OTU47 | Thaumarchaeota | Thaumarchaeota | Thaumarchaeota | Thaumarchaeota | Thaumarchaeota | 33 | 1 |
|  | OTU68 | Crenarchaeota | Crenarchaeota | Crenarchaeota | Crenarchaeota | Crenarchaeota | 33 | 1 |
|  | OTU116 | Thaumarchaeota | Thaumarchaeota | Thaumarchaeota | Thaumarchaeota | Thaumarchaeota | 33 | 1 |
|  | OTU119 | Thaumarchaeota | Thaumarchaeota | Thaumarchaeota | Thaumarchaeota | Thaumarchaeota | 33 | 1 |
| Inorganic fertilizer group | OTU157 | Crenarchaeota | Thaumarchaeota | Thaumarchaeota | Thaumarchaeota | Thaumarchaeota | 42 | 2 |
|  | OTU2 | Thaumarchaeota | norank | Nitrososphaerales | Nitrososphaeraceae | Nitrososphaera | 41 | 2 |
|  | OTU140 | Thaumarchaeota | Thaumarchaeota | Nitrososphaerales | Nitrososphaeraceae | Nitrososphaera | 41 | 2 |
|  | OTU84 | Thaumarchaeota | norank | Nitrososphaerales | Nitrososphaeraceae | Nitrososphaera | 40 | 2 |
|  | OTU88 | Thaumarchaeota | Thaumarchaeota | Nitrososphaerales | Nitrososphaeraceae | Nitrososphaera | 40 | 2 |
|  | OTU124 | Crenarchaeota | Crenarchaeota | Crenarchaeota | Crenarchaeota | Crenarchaeota | 40 | 2 |
|  | OTU141 | Thaumarchaeota | Thaumarchaeota | Nitrososphaerales | Nitrososphaeraceae | Nitrososphaera | 40 | 2 |
|  | OTU21 | Thaumarchaeota | Thaumarchaeota | Nitrososphaerales | Nitrososphaeraceae | Nitrososphaera | 39 | 2 |
|  | OTU51 | Thaumarchaeota | Thaumarchaeota | Thaumarchaeota | Thaumarchaeota | Thaumarchaeota | 39 | 2 |
|  | OTU66 | Thaumarchaeota | Thaumarchaeota | Nitrososphaerales | Nitrososphaeraceae | Nitrososphaera | 38 | 2 |
|  | OTU11 | Archaea | Archaea | Archaea | Archaea | Archaea | 37 | 2 |

**Table S3.** Hub analysis in the network of AOA by different fertilizer application (Top 10 degree of OTUs was selected).

**Table S4.** Hub analysis in the network of AOB by different fertilizer application (Top 10 degree of OTUs was selected).

|  | OTU | Phylum | Class | Order | Family | Genus | Degree | Modularity_class |
| --- | --- | --- | --- | --- | --- | --- | --- | --- |
| Organic fertilizer group | OTU10 | Proteobacteria | Betaproteobacteria | Nitrosomonadales | Nitrosomonadaceae | Nitrosospira | 23 | 0 |
|  | OTU46 | Proteobacteria | Betaproteobacteria | Nitrosomonadales | Nitrosomonadaceae | Nitrosospira | 23 | 0 |
|  | OTU6 | Proteobacteria | Betaproteobacteria | Nitrosomonadales | Nitrosomonadaceae | Nitrosospira | 22 | 2 |
|  | OTU19 | Proteobacteria | Betaproteobacteria | Nitrosomonadales | Nitrosomonadaceae | Nitrosomonadaceae | 22 | 0 |
|  | OTU47 | Proteobacteria | Betaproteobacteria | Nitrosomonadales | Nitrosomonadales | Nitrosomonadales | 22 | 0 |
|  | OTU65 | Proteobacteria | Betaproteobacteria | Nitrosomonadales | Nitrosomonadaceae | Nitrosospira | 22 | 0 |
|  | OTU13 | Proteobacteria | Betaproteobacteria | Nitrosomonadales | Nitrosomonadaceae | Nitrosospira | 21 | 0 |
|  | OTU14 | Proteobacteria | Betaproteobacteria | Nitrosomonadales | Nitrosomonadaceae | Nitrosospira | 21 | 0 |
|  | OTU54 | Proteobacteria | Betaproteobacteria | Nitrosomonadales | Nitrosomonadaceae | Nitrosospira | 21 | 2 |
|  | OTU58 | Proteobacteria | Betaproteobacteria | Nitrosomonadales | Nitrosomonadaceae | Nitrosospira | 21 | 0 |
| Inorganic fertilizer group | OTU78 | Proteobacteria | Betaproteobacteria | Nitrosomonadales | Betaproteobacteria | Betaproteobacteria | 27 | 5 |
|  | OTU28 | Proteobacteria | Betaproteobacteria | Nitrosomonadaceae | Nitrosomonadaceae | Nitrosomonadaceae | 26 | 1 |
|  | OTU31 | Proteobacteria | Betaproteobacteria | Proteobacteria | Proteobacteria | Proteobacteria | 26 | 5 |
|  | OTU33 | Proteobacteria | Proteobacteria | Proteobacteria | Proteobacteria | Proteobacteria | 26 | 4 |
|  | OTU36 | Proteobacteria | Betaproteobacteria | Nitrosomonadales | Nitrosomonadales | Nitrosomonadales | 26 | 4 |
|  | OTU6 | Proteobacteria | Betaproteobacteria | Nitrosomonadales | Nitrosomonadaceae | Nitrosospira | 25 | 4 |
|  | OTU50 | Proteobacteria | Proteobacteria | Proteobacteria | Proteobacteria | Proteobacteria | 25 | 5 |
|  | OTU69 | Proteobacteria | Betaproteobacteria | Nitrosomonadales | Nitrosomonadaceae | Nitrosospira | 25 | 4 |
|  | OTU88 | Proteobacteria | Proteobacteria | Proteobacteria | Proteobacteria | Proteobacteria | 25 | 4 |
|  | OTU27 | Proteobacteria | Proteobacteria | Proteobacteria | Proteobacteria | Proteobacteria | 24 | 4 |

**Table S5.** Organic and inorganic fertilizer application program for black soil in this study.

| Treatments | N (kg/hm^2^· y) | P_2_O_5_ (kg/hm^2^· y) | K_2_O (kg/hm^2^· y) |
| --- | --- | --- | --- |
| CK | 0 | 0 | 0 |
| N1 | 75 | 150 | 75 |
| N2 | 150 | 150 | 75 |
| M | 104 | 117 | 165 |

Note: CK, N1, N2 and M means without fertilizer, low level inorganic fertilizer,high level inorganic fertilizer and organic fertilizer in black soil.

**Table S6** Statistics of samples for AOA and AOB followed by different fertilizer.

| Sample | AOA | | | AOB | | |
| --- | --- | --- | --- | --- | --- | --- |
|  | Valid | Trimed | Percent | Valid | Trimed | Percent |
| CK_1 | 18448 | 16258 | 88.13% | 18130 | 15646 | 86.30% |
| CK_2 | 14393 | 12750 | 88.58% | 16283 | 14483 | 88.95% |
| CK_3 | 18813 | 16944 | 90.07% | 17712 | 15937 | 89.98% |
| CK_4 | 17950 | 15849 | 88.30% | 17057 | 15341 | 89.94% |
| CK_5 | 13439 | 12046 | 89.63% | 15965 | 14260 | 89.32% |
| M_1 | 19206 | 17117 | 89.12% | 16540 | 13867 | 83.84% |
| M_2 | 19359 | 16784 | 86.70% | 15543 | 13471 | 86.67% |
| M_3 | 18235 | 16256 | 89.15% | 15459 | 13200 | 85.39% |
| M_4 | 18030 | 16070 | 89.13% | 15320 | 13132 | 85.72% |
| M_5 | 18825 | 16889 | 89.72% | 13996 | 12092 | 86.40% |
| N1_1 | 17959 | 16699 | 92.98% | 15308 | 13142 | 85.85% |
| N1_2 | 17277 | 15935 | 92.23% | 11889 | 10510 | 88.40% |
| N1_3 | 17245 | 16004 | 92.80% | 10207 | 8885 | 87.05% |
| N1_4 | 14683 | 13774 | 93.81% | 11659 | 10180 | 87.31% |
| N1_5 | 18082 | 16778 | 92.79% | 12481 | 10821 | 86.70% |
| N2_1 | 17534 | 16561 | 94.45% | 12871 | 11199 | 87.01% |
| N2_2 | 19476 | 18194 | 93.42% | 13388 | 11651 | 87.03% |
| N2_3 | 18213 | 17089 | 93.83% | 15292 | 13247 | 86.63% |
| N2_4 | 18044 | 16986 | 94.14% | 14597 | 12675 | 86.83% |
| N2_5 | 19225 | 17902 | 93.12% | 13420 | 11599 | 86.43% |

**Table S7 Statistics of trimed sequences** for AOA and AOB followed by different fertilizer.

| Samples | AOA | | | AOB | | |
| --- | --- | --- | --- | --- | --- | --- |
|  | Sequences | Bases (bp) | Average Length (bp) | Sequences | Bases (bp) | Average Length (bp) |
| CK_1 | 16258 | 6954739 | 427.77 | 15646 | 6952287 | 444.35 |
| CK_2 | 12750 | 5480699 | 429.86 | 14483 | 6438240 | 444.54 |
| CK_3 | 16944 | 7288835 | 430.17 | 15937 | 7077855 | 444.11 |
| CK_4 | 15849 | 6814872 | 429.99 | 15341 | 6826999 | 445.02 |
| CK_5 | 12046 | 5166578 | 428.90 | 14260 | 6327505 | 443.72 |
| M_1 | 17117 | 7384318 | 431.4 | 13867 | 6139599 | 442.75 |
| M_2 | 16784 | 7159415 | 426.56 | 13471 | 5980191 | 443.93 |
| M_3 | 16256 | 6998983 | 430.55 | 13200 | 5840441 | 442.46 |
| M_4 | 16070 | 6902511 | 429.53 | 13132 | 5827618 | 443.77 |
| M_5 | 16889 | 7247265 | 429.11 | 12092 | 5367489 | 443.89 |
| N1_1 | 16699 | 7212420 | 431.91 | 13142 | 5781466 | 439.92 |
| N1_2 | 15935 | 6878985 | 431.69 | 10510 | 4672025 | 444.53 |
| N1_3 | 16004 | 6845899 | 427.76 | 8885 | 3931560 | 442.49 |
| N1_4 | 13774 | 5904800 | 428.69 | 10180 | 4474922 | 439.58 |
| N1_5 | 16778 | 7179613 | 427.92 | 10821 | 4750946 | 439.05 |
| N2_1 | 16561 | 7162653 | 432.50 | 11199 | 4847306 | 432.83 |
| N2_2 | 18194 | 7900769 | 434.25 | 11651 | 5047560 | 433.23 |
| N2_3 | 17089 | 7443129 | 435.55 | 13247 | 5709157 | 430.98 |
| N2_4 | 16986 | 7397647 | 435.51 | 12675 | 5471469 | 431.67 |
| N2_5 | 17902 | 7774809 | 434.30 | 11599 | 5023014 | 433.06 |
